# A bio-economic simulation study on the association between key performance indicators and pluck lesions in Irish farrow-to-finish pig farms

**DOI:** 10.1186/s40813-020-00176-w

**Published:** 2020-12-09

**Authors:** Julia Adriana Calderón Díaz, Maria Rodrigues da Costa, Laurence Shalloo, Jarkko K. Niemi, Finola Catherine Leonard, Daniel Crespo-Piazuelo, Josep Gasa, Edgar García Manzanilla

**Affiliations:** 1 Pig Development Department, Teagasc Animal and Grassland Research and Innovation Centre, Moorepark, Fermoy, Co. Cork, Ireland; 2 School of Veterinary Medicine, University College Dublin, Dublin, Ireland; 3 Epidemiology research unit, Scotland’s rural College (SRUC), Inverness, Scotland IV2 5NA UK; 4 Livestock Production Systems, Teagasc Animal and Grassland Research and Innovation Centre, Moorepark, Fermoy, Co. Cork, Ireland; 5 Natural Resources Institute Finland (LUKE), Kampusranta 9, 60320 Seinäjoki, Finland; 6 Departament de Ciència Animal I dels Aliments, Facultat de Veterinaria, Universitat Autònoma de Barcelona, Barcelona, Spain

**Keywords:** Economic modelling, Lung scars, Pig production systems, Pleurisy, Regression trees, Stochastic budgeting

## Abstract

**Background:**

Pluck lesions are associated with decreased performance in grower-finisher pigs, but their economic impact needs to be further investigated. This study aimed to identify the main pluck lesions and the cut-off value for their prevalence, associated with changes in average daily gain (ADG) during the wean-to-finish period, to simulate their effects on economic performance of farrow-to-finish farms. Pigs (*n* = 162 ± 51.9 per farm) from 56 farrow-to-finish farms were inspected at slaughter and the prevalence of enzootic pneumonia-like lesions, pleurisy, lung scars, abscesses, pericarditis, and liver milk spots was estimated. For each farm, annual performance indicators were obtained. Regression trees analysis (RTA) was used to identify pluck lesions and to estimate cut-off values for their prevalence associated with changes in ADG. Different scenarios were simulated as per RTA results and economic and risk analyses were performed using the Teagasc Pig Production Model. Risk analysis was performed by Monte Carlo sampling using the Microsoft Excel add-in *@Risk* with 10,000 iterations.

**Results:**

Pleurisy and lung scars were the main lesions associated with changes in ADG. Three scenarios were simulated based on RTA results: a 728 sow farrow-to-finish farm with prevalence of i) pleurisy < 25% and lung scars < 8% (LPLSC; ADG = 760 g); ii) pleurisy < 25% and lung scar ≥8% (LPHSC; ADG = 725 g) and iii) pleurisy ≥25% (HP; ADG = 671 g). The economic analysis showed increased feed and dead animals for disposal costs, and lower sales in the HP and LPHSC scenarios than in the LPLSC scenario; thereby reducing gross margin and net profit. Results from the risk analysis showed lower probability of reaching any given level of profit in the HP scenario compared with the LPHSC and LPLSC scenarios.

**Conclusion:**

Under the conditions of this study, higher prevalence of pleurisy and lung scars were associated with decreased ADG during the grower-finisher period and with lower economic return in the simulated farms. These results highlight the economic benefits and importance of preventing and/or controlling respiratory disease.

## Background

Assessment of pluck lesions can be used as a tool for on-farm disease surveillance as these lesions reflect respiratory disease affecting pigs [1] and a high prevalence of pluck lesions could be indicative of farms with higher proportion of diseased animals [2]. Lesions in the lungs, heart, pleura and liver are the most important ones observed during *post-mortem* examinations at the abattoir [3]. They represent lesions caused by respiratory pathogens and parasite infestations that contribute to reduced performance [4–7] during the grower-finisher period. Pneumonia and pleurisy are the most commonly observed lung lesions at the abattoir [8–10] and they are associated with an increased likelihood of observing other lung lesions such as abscesses and scars [3, 11]. Further, infestation by *Ascaris suum*, as the larvae migrate through the liver and lungs, may cause both direct damage to the lungs and affect the immune response to pathogens such as *Mycoplasma hyopneumoniae* [12]*,* which could potentially increase prevalence of pneumonia lesions. Lesions observed at the abattoir are those developed closer to slaughter, except for scars [13]. Lesions occurring earlier in life can resolve [13, 14] and thus, pluck lesions could underestimate the true prevalence of lung, heart and liver lesions during the production cycle. However, it is possible that even when lesions resolved, performance is reduced in affected pigs. For instance, pluck lesions are associated with higher mortality rates [2] and reduced average daily gain (ADG, [7, 15, 16]) during the grower-finisher period and longer time to reach target slaughter weight [13] resulting in reduced feed conversion efficiency [7, 17]. Additionally, pluck lesions are associated with carcass yield losses due to condemnations or trimmings [18].

While respiratory disorders are known to cause substantial economic losses to pig farms (e.g. [19, 20]), there is no information regarding hierarchy of the effects of the different pluck lesions and cut-off values for their prevalence that could be used for decision making to minimize adverse effects on animal performance, and farm profitability. Currently, farmers and veterinarians do not use objective quantitative methods to decide at which prevalence a disease becomes a problem. The hierarchy and cut-off values for the prevalence of different lesions that producers and veterinarians see as critical for intervention could be different than a cut-off value obtained using evidence-based methods in a population of farms. An objectively estimated hierarchy and cut-off values for the prevalence of pluck lesions for interventions could aid producers and veterinary practitioners to maximise benefits on their farms.

Although pluck lesions are associated with decreased performance [7, 13, 15–17], only few studies have conducted economic analyses. Jäger et al. [21] quantified the economic impact of pleurisy in British pig herds and estimated a cost of £5 (approximately €5.8) per pig produced. Their calculations were based on industry standard costs associated with 6% increase in mortality during the grower-finisher period, a prevalence of pleurisy of 20%, reduction in ADG of 50 g and reduction of feed conversion efficiency of 0.1. Ferraz et al. [6] estimated a cost of US$6.55 (approximately €5.5) per pig produced considering a reduction in ADG of 27 g associated with lesions (pneumonia and pleurisy) in ≥15% of the lungs compared with pigs with no lesions. Doing these calculations is not easy as there are other variables such as market prices fluctuations and the complex interrelationships between different aspects of production to consider when using real-world farm-level data. Using bio-economic stochastic modelling approaches can provide opportunities to consider all factors influencing the economic performance and be flexible when analysing their impact. Indeed, bio-economic models describe the associations between different biological and financial components of farming systems [22]. Also, by incorporating stochasticity, bio-economic models allow to account for the uncertain impact of pathogens and parasites on animal performance and of market conditions on production costs and farm profitability. This study aimed to 1) identify the main pluck lesions associated with changes in ADG during the wean-to-finish period, 2) estimate the cut-off values for the prevalence of these lesions that maximize effects on ADG in the studied population, and 3) simulate the economic performance of a farrow-to-finish farm by parameterising a previously constructed stochastic bio-economic model with production effects associated with the cut-off values for the prevalence of the pluck lesions identified in objective one.

## Methods

### Farm selection, prevalence of pluck lesions and performance indicators

This study is part of a larger project investigating respiratory disease on Irish pig farms, associated risk factors, and the relationship with performance, welfare and antimicrobial use. The farms participating in this study were part of the Teagasc e-Profit Monitor. Teagasc is the Irish Authority for Agriculture and Food Development, a semi-state organisation responsible for providing integrated research, advisory and education/training services for the agriculture and food industry in Ireland. The Teagasc e-Profit Monitor is an online financial analysis system for assessing farm profitability which contains biological and economic records. Farms (*n* = 107) providing data to the Teagasc e-Profit Monitor in the year 2017 were contacted directly by telephone or via their Teagasc pig advisor specialist and invited to participate in the study. A total of 56 farmers voluntarily agreed to participate (52.3% participation rate). All 56 farms participating in the study were farrow-to-finish farms with weekly farrowing batches which is the predominant pig production system in Ireland [23].

Upon written consent from the farmer, a visit to the abattoir was schedule to inspect pluck lesions in a batch of pigs per farm. The absence of respiratory disease outbreaks (i.e. an increase in mortality or clinical signs that resulted in a major change in medication or vaccination) was confirmed during the call to schedule the abattoir visit. In total, 9254 pigs (162 ± 51.9 pigs; range 55 to 308) were inspected at slaughter for lesions in the lungs, heart and liver by a single trained observer from November 2017 to April 2018. During the data collection period, the observer was blind to herd positive or negative status with regards to four of the most common respiratory pathogens (i.e. *Actinobacillus pleuropeneumoniae, Mycoplasma hyopneumoniae,* porcine reproductive and respiratory syndrome virus and swine influenza virus) involved in the porcine respiratory disease complex (PRDC, [24, 25]) and, that are associated with the development of pluck lesions [26].

Lung were removed from the carcass by abattoir personnel and assessed in the evisceration line for the presence of enzootic pneumonia-like lesions, dorso-caudal pleurisy, lung scars (i.e. healing indicative of pneumonic lesions which developed earlier in the pig’s life) and abscesses. Pericarditis and liver milk spots (i.e. presence of white spots in the liver suggestive of trans-hepatic migration of the larvae of *Ascaris suum*) were also recorded. All pluck lesions were scored using the Ceva Lung Program app (CEVA Santé Animale, Libourne, France) to facilitate data recording. Enzootic pneumonia-like lesions were assessed according to the method described by Madec and Derrien [27] with the overall surface affected averaged accounting for lobe weights [28]. Pleurisy was scored in the dorso-caudal lobes using a modified version of the Slaughterhouse Pleuritis Evaluation System (SPES, [29]) in a 4-point scale where 0 = no pleurisy; 2 = focal lesions in one lobe; 3 = bilateral adhesions or monolateral lesions affecting more than 1/3 of the diaphragmatic lobe and 4 = extensive lesions affecting more than 1/3 of both diaphragmatic lobes. Lungs that remained attached to the chest wall and were not removed from the carcass were not scored. Pleurisy scores ≥2 were used to calculate the prevalence of pleurisy for each farm. Lung scars and abscesses, pericarditis and liver milk spots were recorded as present or absent.

For each participating farm, annual mean performance indicators required to parameterise the bio-economic model [30] were retrieved from the Teagasc e-Profit monitor for the year 2017. Performance indicators included farrowing rate, litters per sow per year, average number of piglets born alive per litter, culling rate, mortality rates for different production stages, ADG, live weight at sale and dressing percentage.

### Associations between pluck lesions and production parameters

Regression tree [31] analysis was used to define the main lesions associated with ADG during the grow-finisher period and the cut-off value for their prevalence resulting in partitions showing maximum differences in ADG. Average daily gain was used as the main output variable based on previous reports in the scientific literature regarding decreased performance associated with presence of pluck lesions (e.g. [4, 7]) and on previous results from our research group. Regression tree analysis is a non-parametric statistical technique based on recursive partitioning analysis [32] that selects those variables and their interactions that are most important in predicting the outcome variable by calculation of their relative importance [33]. In other words, a regression tree is a hierarchically organized structure [34]. There are several advantages to using regression tree analysis including that it does not require the data to be linear, and thus, accommodating multi-collinearity among predictors [33]; predictors can be continuous or categorical and it accounts for multiple interactions among predictors [35]. Additionally, the results are presented in a way that is easy to interpret by people not (very) familiar with statistical analyses.

Contrary to “classical” regression methods, regression tree analysis does not develop a prediction equation but rather data are partitioned into subsets with homogeneous values of the dependent variable [36, 37]. In general terms, the recursive partitioning process can be generalised to

$$ \hat{f}(X)=\sum \limits_{m=1}^n{c}_mI\Big\{\left({X}_1,{X}_{2,}\right)\in {R}_{m\Big\}} $$

indicating there is a continuous response variable *Y* and inputs X_1_ and X_2_. *c*_*m*_ are constants; *I* is an indicator function returning 1 if its argument is true and 0 otherwise and the recursive portioning resulting in *R*_*m*_ number of subsets. Each subset is created based on the regression tree identifying the predictor and split value that partitions the data into two regions where the overall sum of squares errors are minimised [37].

The recursive portioning process is done in a top-down fashion where the portioned done earlier in the tree will not change based on later partitions [38]. A regression tree starts with a root node containing all the subjects; this is called a *parent node*. Every value of each predictor variable is considered as a potential split. The optimal split is selected as the (cut-off) value of the predictor variable that forms binary groups that are most different with respect to the dependent variable [36]. This results in the *parent node* being split into two *child nodes* (or branches) which in turn can be split into additional nodes [35]. The procedure continues through each node of the tree until a stopping rule is reached. At the point that no further split is made, a *terminal node* (or leaf) is created. The average value of the dependent variable is estimated among the subjects within each node.

Regression tree analysis was done using the *rpart* package [39] in *R* v3.4.2 [40]. It included ADG during the grower-finisher period as outcome variable and the prevalence of the six recorded pluck lesions as explanatory variables. The stopping criterion was a minimum of 10 farms being required to create a branch and/or a leaf. This minimum of 10 farms was estimated based on a minimum difference in ADG of at least 40 g between regression tree partitions used to calculate sample size for a t-test for independent samples in PROC POWER of SAS v9.4 (SAS Inst. Inc., Cary, NC).

After building the regression tree, it was pruned to find an optimal size and avoid over-fitting of the data by using a cost complexity parameter (α) that penalises the minimization of the sum of squares errors for the number of terminal nodes on the regression tree (T)

$$ Minimise\left\{ SSE+\alpha \left|T\right|\right\} $$

for a given value of α, the smallest pruned tree that has the lower penalised error is found. If the cost of adding another variable to the regression tree from the current node is above the value of cost complexity parameter, then tree building does not continue. The *printcp*() function in *rpart* [39] was used to identify the cost complexity parameter having the least cross-validated error and use it to prune the regression tree. ANOVA test was performed between each binary partition to determine whether the groups created were statistically different from each other using an alpha of 0.05. Following this, group averages were calculated for performance indicators required to parameterise a bio-economic model [30] that was then used for the economic analysis of the different scenarios describe below.

### Bio-economic simulation

#### Economic analysis

The Teagasc Pig Production model (TPPM; Calderón Díaz et al. [30]) was used to simulate annual economic performance of a farrow-to-finish pig farm using information on the association between key performance indicators and the cut-off values for the main pluck lesions identified during the regression tree analysis. The TPPM is a stochastic budgetary bio-economic simulation model developed in Microsoft Excel. The TPPM is representative of the predominant intensive farrow-to-finish pig producing farms in Ireland with weekly farrowing batches, and several animal categories with different infrastructure and feeding practices for each production stage [30]. The TPPM integrates biological, physical and technical parameters and economic analysis. It allows the user to investigate the impact of changes in pig production systems on farm performance and profitability. First, the TPPM simulates weekly numbers of maiden gilts (24 to 32 weeks of age), gestating sows (≥ 32 weeks of age), lactating sows (≥48 weeks of age), weaner pigs in stage 1 (c. 7 kg of body weight on transfer to this stage), weaner pigs in stage 2 (c. 19 kg of body weight on transfer to this stage), and finisher pigs (c. 38 kg of body weight on transfer to this stage) based on biological inputs such as herd size, conception and farrowing rate, number of litters per sow per year, number of piglets born alive per litter and mortality rate for each production stage.

Then, to simulate animal growth during the wean-to-finish period, the TPPM includes the Gompertz growth function [41] using the formula *BW* = *W*_0_ exp[*μ*_0_(1 − *e*^−*Dt*^)/*D*]; where **BW** = body weight; ***W***_**0**_ = the value of the growth function at age 0; ***μ***_**0**_ = logarithm of the relative growth rate at age 0 and ***D*** = slope of the logarithm of the relative growth rate. Dietary nutritional requirements (i.e. energy, amino acids and minerals) vary for each production stage and are estimated following the recommendations from the National Research Council (NRC) Nutrient Requirements of Swine [42]. For each production stage, wheat-barley-soya-based diets were formulated within the TPPM to meet or exceed NRC [42] requirements. All diets are representative of common feeding practice in Irish pig farms. Daily energy demand and feed intake are estimated following the NRC [42] equations for estimating nutrient requirements for weaner-finisher pigs according to their estimated BW obtained from the Gompertz growth curve (for more information please refer to Calderón Díaz et al. [30]).

Biological performance is then combined with management practices including reproductive management, labour, herd healthcare plan and infrastructure available at the farm. Costs associated with management practices are also included in the TPPM. Other costs such as the annual subscription to the Environmental Protection Agency, transport costs per pig to the abattoir, long term bank loans payments, and monthly feedstuff prices are also included. In the TPPM, the only source of income is the sale of culled sows and finisher pigs. Cold carcass weight is calculated by multiplying body weight at sale by the dressing percentage. Average monthly price per kg of meat is used to calculate income per pig. For more information regarding the assumptions of the bio-economic model please refer to Calderón Díaz et al. [30]*.*

Physical outputs from the TPPM include annual number of pig produced, annual number of kg of meat sold and annual feed usage during the different production stages. Financial outputs from the TPPM include variable and fixed costs, gross income, net profit, an annual cash flow budget, annual profit and loss account, and annual balance sheet. Variable and fixed costs and sales are simulated based on current market costs and prices. The estimated annual gross income and net profit is presented on a total farm basis, as well as per pig produced and per kg of meat sold.

We simulated a 728 farrow-to-finish farm with weekly farrowing batches for an entire year. This herd size corresponds to the mean herd size in Ireland for the year 2017 [43]. Three different scenarios were simulated based on results from the regression tree analysis. Information on mean farrowing rate, litters per sow per year, number of piglets born alive per litter, sow culling and sow mortality rate, mortality rates during the weaner stage one, weaner stage two and finisher stage and dressing percentage were calculated for each scenario based on farm record from the Teagasc e-profit monitor for the year 2017 (Table 1). In the simulated farm, all animal categories were included (i.e., piglets, weaner and finisher pigs, maiden gilts, pregnant and lactating sows and boars for heat detection). The number of gilts, gestating and lactating sows as well as number of piglets, weaners and finisher pigs were calculated each week within the TPPM based on the mortality rates for each production stage and varied for each scenario. For all the scenarios, all pigs were vaccinated for porcine circovirus type 2 and *Mycoplasma hyopneumoniae* at weaning (i.e. 28 days of age) with a single dose. Also, maiden gilts and lactating sows were vaccinated for *Erysipelothrix rhusiopathiae* and porcine parvovirus as per normal practice in Irish pig farms. Prices for the vaccines were obtained from a major veterinary distributor in Ireland. In Ireland, only 1.8% of farms vaccinate for *Actinobacillus pleupneumoniae* and thus, we did not include this vaccination in the simulation. Four veterinarian visits per year at a cost of €300 each were considered for the economic analysis as per usual practice.

**Table 1:** Biological parameters^a^ obtained from the Teagasc e-Profit monitor used to parameterised the Teagasc Pig Production Model [30] to simulate^b^ effects associated with different prevalence of pleurisy and lung scars on slaughter pigs on farm performance and profitability

Parameter	LPLSC^**c**^			LPHSC^**d**^			HP^**e**^		
	Min	Mean	Max	Min	Mean	Max	Min	Mean	Max
Farrowing rate, %	81	89.3	95.7	73	88.2	96.1	81.8	87.9	91.2
Litters per sow per year	2.09	2.30	2.43	2.11	2.28	2.50	2.24	2.33	2.46
Number born alive piglets per litter	12.4	13.7	15.2	11.6	13.6	15.3	12.8	13.4	14.0
Sow culling rate, %	34.3	46.9	58.0	37.8	51.1	63.9	39.0	47.0	54.9
Sow mortality, %	2.3	4.2	9.3	1.8	5.4	9.2	3.2	4.7	9.7
Piglet mortality, %	6.8	10.7	14.3	5.7	10.8	15.9	5.6	9.9	14.0
Weaner mortality, %	0.5	2.1	6.8	0.9	2.9	8.9	1.5	3.7	7.0
Finisher mortality, %	0.9	1.7	3.3	1.0	2.1	4.1	1.3	2.3	3.0
Dressing, %	75.1	76.2	78.1	74.8	76.2	77.4	76.0	76.8	77.8

^a^Mean values were used for each scenario during the economic analysis. The range of values was used to fit probability distributions for the stochastic simulation using Monte Carlo sampling in the Microsoft Excel add-in *@Risk* [44]

^b^A 728 sow farrow-to-finish farm with weekly farrowing batches was simulated to represent three different scenarios

^c^Scenario 1: a farrow-to-finish farm with prevalence of pleurisy < 25% and prevalence of lung scars < 8% with a wean-to-finish average daily gain (ADG) of 760 g and reaching target slaughter weight at 24 weeks of age

^d^Scenario 2: a farrow-to-finish farm with prevalence of pleurisy < 25% and prevalence of lung scars ≥8% (LPHSC) with an ADG of 725 g and reaching target slaughter weight at 25 weeks of age

^e^Scenario 3: a farrow-to-finish farm with prevalence of pleurisy ≥25% (HP) with and ADG of 671 g and reaching target slaughter weight at 26 weeks of age

**Fig. 1:**
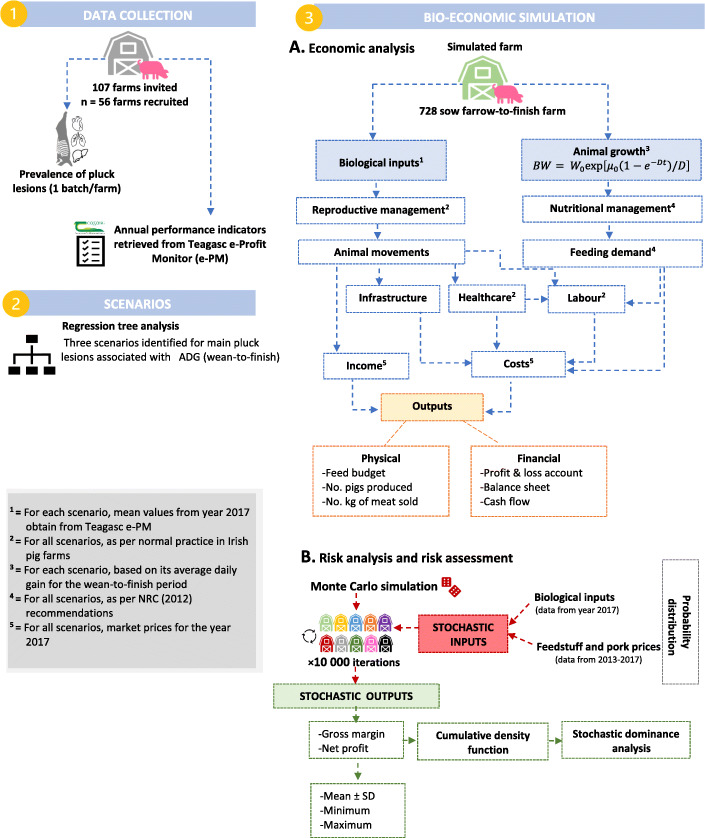
Flow diagram describing data sources and bio-economic modelling process followed to simulate effects associated with different prevalence of pleurisy and lung scars on slaughter pigs on farm performance and profitability. Three scenarios were simulated for a 728 sow farrow-to-finish farm

In all scenarios, pigs were weaned at 28 days of age and 7 kg of body weight. Animal growth during the wean-to-finish period was simulated to represent the different ADG rates obtained from the regression tree analysis. However, all pigs were slaughtered at 110.8 kg of body weight which was the average body weight at sale in Irish farms in the year 2017 [43]. Carcass condemnations associated with pluck lesions were not considered in the simulation as no data were available; however, a 1.5% condemnation rate was used for all scenarios. Monthly feedstuff and pork prices (per kg) for the year 2017 were obtained from the Teagasc e-Profit monitor and did not vary between scenarios. In all scenarios, diet formulation and feed intake was calculated as a function of body weight as previously explained. Infrastructure (e.g. number of pig spaces available in each production stage, depreciation), management practices, labour requirements, capital investment did not differ between scenarios. A flow diagram describing data sources and the bio-economic simulation process followed in this study is presented in Fig. 1.

#### Risk analysis and risk assessment

Risk analysis (i.e. the identification of possible outcomes) and risk assessment (i.e. estimation of probabilities and the economic impacts that result from the corresponding outcomes [45]) were implemented through Monte Carlo sampling using the Microsoft Excel add-in *@Risk* [44]. Risk analysis and risk assessment provide information that can be used as the basis for evaluating different scenarios allowing for better decision making under uncertainty. Reproductive performance, mortality rates, feed prices and pork market conditions are among the most important variables affecting profitability in pig farms. To account for uncertainty and variation in biological inputs, feed costs and carcass prices, they were included as stochastic input variables during the risk analysis. Estimates for feedstuff and pork prices (per kg) were generated based on data recorded on the Teagasc pig e-Profit monitor between the years 2013 to 2017. By including historical data, we provide the TPPM with a range of values that better reflect market conditions as feedstuff and pork prices can experience drastic changes in relatively short periods of time. Estimates for biological parameters were generated for each variable for each scenario based on the value range obtained from the Teagasc e-profit monitor for the year 2017 (Table 1). For each stochastic variable, the distribution fitting function from *@Risk* was used to determine their appropriate distribution for the Monte Carlo simulation. Spearman’s rank correlations were estimated in PROC CORR of SAS v9.4 (SAS Inst. Inc., Cary, NC) to account for possible co-variation between stochastic input variables, and they were included during the Monte Carlo simulation. Gross margin and annual net profit were set as the stochastic output variables. A total of 10,000 iterations were run during the Monte Carlo simulation. At each iteration, all stochastic input variables varied simultaneously by randomly sampling a new set of values for each variable from their corresponding distributions. Additionally, gross margins and net profit were calculated for each iteration. Finally, annual mean gross margin and net profit and the variation of results around the mean were reported. In addition, stochastic dominance analysis, which compared distributions of income between scenarios, was carried out by performing pairwise comparisons of income distributions for different scenarios being considered by inspecting their cumulative distribution function (CDF) curve. The CDF describes the probability that a variable *X* is less than or equal to *x:*

$$ F(x)=P\left(X\le x\right)\kern1.75em for\  all\ x $$

Stochastic dominance is a partial order of random variables. Scenarios with a CDF further to the right are preferred and thus, the income distribution that exceeds the other, at any level, is stochastically dominant indicating lower economic risk and allowing the identification of the preferred scenario [46]. When two alternatives *A* and *B*, each with a probability distribution of outcomes *x* [defined by the cumulative probability of *F*_*A*_(*x*) and *F*_*B*_(*x*)] are compared, *A* first-order stochastically dominates *B* if *F*_*A*_(*x*) ≤ *F*_*B*_(*x*), for all *x* and there is a strong inequality in at least one point of the distribution. Graphically, the CDF of *A* must always lie below and to the right of the cumulative probability of *B*. Second-order stochastic dominance is examined by comparing the integrals of CDFs (i.e. area under CDF): *A* second-order stochastically dominates *B* if

$$ {\int}_{-\infty}^x{F}_A(x) dx\le {\int}_{-\infty}^x{F}_B(x) dx $$

for all *x* with a strict inequality for some range of the distribution. This implies that in a subset of distribution, the dominating alternative *A* may not lead to a better outcome than the dominated alternative *B*. Graphically, the CDF of the dominating scenario *A* is still further to the right for the most part of the CDF and more predictable than the dominated alternative *B*, but not for the entire distribution.

## Results

### Regression tree analysis and selection of simulated scenarios

**Fig. 2:**
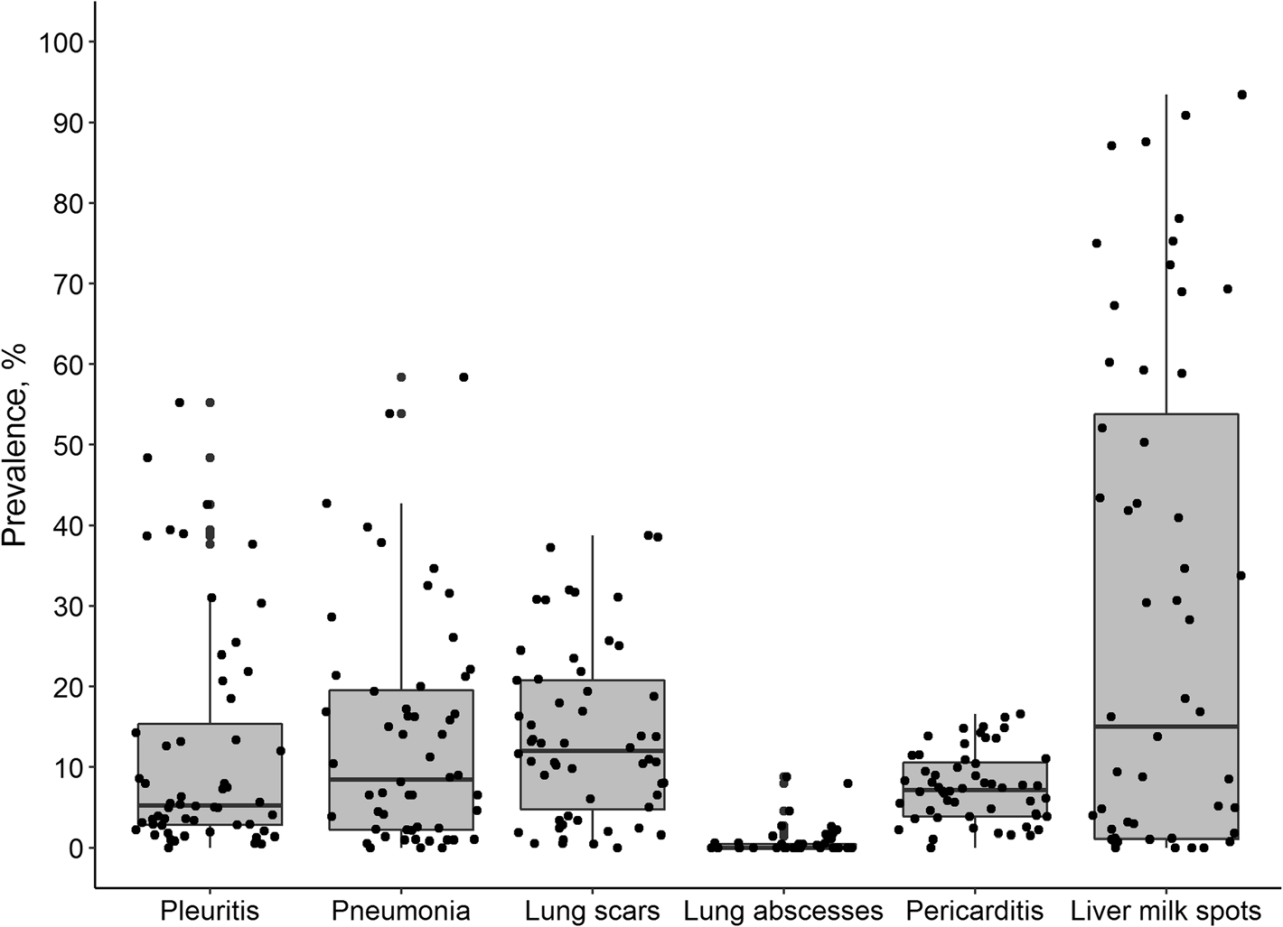
Box and whisker plots for the prevalence (%) of pluck lesions (pleurisy, enzootic pneumonia-like lesions, lung scars and abscesses, pericarditis and liver milk spots) in finisher pigs from 56 farrow-to-finish farms. Each dot represents a farm

**Fig. 3:**
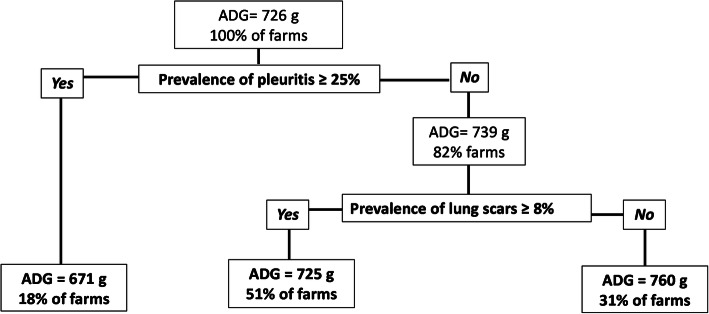
Regression trees for average daily gain (ADG) where the prevalence of pleurisy, pneumonia, scars, pericarditis, abscesses, and liver milk spots were included as predictor variables. The cut-off value of the prevalence of pleurisy and lung scars showed the best division of farms with ADG (g). The percentage of farms in each node/leaf is given for the different groups

The prevalence of each studied pluck lesion in 56 farms included in the study is shown in Fig. 2 while results for the regression tree analysis are shown in Fig. 3. The main pluck lesion associated with changes in ADG was dorso-caudal pleurisy followed by lung scars. The associated cut-off values for these lesions were 25% prevalence of dorso-caudal pleurisy and 8% prevalence of lung scars. Thus, three different scenarios representing different extents of lung lesions on the farms were defined based on these cut-off values:

- Scenario 1: A farrow-to-finish farm with prevalence of pleurisy < 25% and prevalence of lung scars < 8% (LPLSC). Assuming a weaning weight of 7 kg at 4 weeks of age and a slaughter weight of 110.8 kg, on average, pigs will reach target slaughter weight at 24 weeks of age. This was the average age at slaughter of Irish pigs as per data from the e-Profit monitor. This scenario was parameterised with biological inputs originating from 17 farms with mean prevalence of pleurisy 3.9% ± 4.94 and mean prevalence of lung scars 2.8% ± 3.32.
- Scenario 2: A farrow-to-finish farm with prevalence of pleurisy < 25% and prevalence of lung scars ≥8% (LPHSC). Assuming a weaning weight of 7 kg at 4 weeks of age as and a slaughter weight of 110.8 kg, on average, pigs will reach target slaughter weight at 25 weeks of age. This scenario was parameterised with biological inputs originating from 29 farms with mean prevalence of pleurisy 7.5% ± 6.17 and mean prevalence of lung scars 18.7% ± 8.34.
- Scenario 3: A farrow-to-finish farm with prevalence of pleurisy ≥25% (HP). Assuming a weaning weight of 7 kg at 4 weeks of age and a slaughter weight of 110.8 kg, on average, pigs will reach target slaughter weight at 26 weeks of age. This scenario was parameterised with biological inputs originating from 10 farms with mean prevalence of pleurisy 38.8% ± 7.55 and mean prevalence of lung scars 19.3% ± 12.1.

### Bio-economic simulation

#### Economic analysis

The farm simulated under the HP scenario had a 10.2 and 6.7% increase in weaner and finisher feed usage, respectively; produced 3.5% fewer pigs and sold 2.5% less meat (Table 2) when compared with the LPLSC scenario farm. Higher feed usage translated into higher feed costs which in turn increased variable costs (Table 3) in the HP farm. Also, variable costs increased in the HP farm due to greater numbers of dead animals for disposal. Sales (i.e. finisher pigs and culled sows) were 2.4% lower in the HP farm than in the LPLSC farm. Additionally, gross margin and net profit decreased in the HP farm by 14.3 and 41.4%, respectively compared with the LPLSC farm. When compared with the LPHSC farm, the HP farm had a 3.7% decrease in weaner feed usage, a 7.2% increase in finisher feed usage and sold 1.1% fewer pigs and 0.3% less meat (Table 2). Higher variable costs were observed in the HP farm due to higher finisher feed costs and a greater number of dead animals for disposal. Additionally, sales were 0.5% lower in the HP farm than in the LPHSC farm (Table 3). Increased variable costs translated into lower gross income (5%) and lower net profit (18.1%) in the HP farm compared with the LPHSC farm.

**Table 2:** Annual physical outputs obtained from the Teagasc Pig Production Model [30] for the simulation^a^ of effects associated with different prevalence of pleurisy and lung scars on slaughter pigs on farm performance and profitability

Output	LPLSC^**b**^	LPHSC^**c**^	HP^**d**^
*Feed usage, ton*			
Gestation feed	540.5	540.5	540.5
Lactation feed	367.8	367.8	367.8
Creep feed	77.3	76.7	76.5
Link feed	142.3	141.2	140.9
Weaner feed	1137.1	1301.7	1253.1
Finisher feed	3164.9	3150.1	3377.6
*Sales*			
No. finisher pigs sold	19,188	18,772	18,564
Meat sold, ton	1595.1	1560.6	1555.4

^a^A 728 sow farrow-to-finish farm with weekly farrowing batches was simulated to represent three different scenarios

^b^Scenario 1: a farrow-to-finish farm with prevalence of pleurisy < 25% and prevalence of lung scars < 8% with a wean-to-finish average daily gain (ADG) of 760 g and reaching target slaughter weight at 24 weeks of age

^c^Scenario 2: a farrow-to-finish farm with prevalence of pleurisy < 25% and prevalence of lung scars ≥8% (LPHSC) with an ADG of 725 g and reaching target slaughter weight at 25 weeks of age

^d^Scenario 3: a farrow-to-finish farm with prevalence of pleurisy ≥25% (HP) with and ADG of 671 g and reaching target slaughter weight at 26 weeks of age

**Table 3:** Comparison of trade profit and loss accounts obtained from the Teagasc Pig Production Model [30] for the simulation^a^ of effects associated with different prevalence of pleurisy and lung scars on slaughter pigs on farm performance and profitability

Item	€/year			€/pig produced			€/kg meat		
	LPLSC^b^	LPHSC^c^	HP^d^	LPLSC	LPHSC	HP	LPLSC	LPHSC	HP
*Sales*									
Finisher pigs	2,582,595	2,526,610	2,518,277	134.6	134.6	135.7	1.62	1.62	1.62
Culled sows	40,972	44,641	41,059	2.1	2.4	2.2	0.03	0.03	0.03
*Total Sales*	2,623,567	2,571,250	2,559,336	136.7	137.0	137.9	1.64	1.65	1.65
*Variable costs*									
Gestation feed	126,460	126,403	126,348	6.6	6.7	6.8	0.08	0.08	0.08
Lactation feed	94,750	94,711	94,674	4.9	5.0	5.1	0.06	0.06	0.06
Creep feed	71,235	70,689	70,506	3.7	3.8	3.8	0.04	0.05	0.05
Link feed	84,540	83,892	83,676	4.4	4.5	4.5	0.05	0.05	0.05
Weaner feed	296,067	338,451	326,804	15.4	18.0	17.6	0.19	0.22	0.21
Finisher feed	753,112	749,258	803,019	39.2	39.9	43.3	0.47	0.48	0.52
Replacement gilts	60,232	68,829	60,219	3.1	3.7	3.2	0.04	0.04	0.04
Dead animal Disposal	9170	11,274	12,213	0.5	0.6	0.7	0.01	0.01	0.01
Health care	41,961	41,689	41,547	2.2	2.2	2.2	0.03	0.03	0.03
Reproduction	37,309	37,309	37,308	1.9	2.0	2.0	0.02	0.02	0.02
Manure handling	16,093	15,866	15,706	0.8	0.8	0.8	0.01	0.01	0.01
Transport	18,183	17,845	17,603	0.9	1.0	0.9	0.01	0.01	0.01
***Total variable costs***	**1,609,112**	**1,656,215**	**1,689,624**	**83.9**	**88.2**	**91.0**	**1.01**	**1.06**	**1.09**
***Gross margin***	**1,014,455**	**915,036**	**869,712**	**52.9**	**48.7**	**46.8**	**0.64**	**0.59**	**0.56**
*Fixed costs*									
Admin and accounting	2500	2500	2500	0.1	0.1	0.1	0.00	0.00	0.00
Electricity, heating and light	81,614	81,614	81,614	4.3	4.3	4.4	0.05	0.05	0.05
Insurance	20,533	20,533	20,533	1.1	1.1	1.1	0.01	0.01	0.01
Repairs	20,533	20,533	20,533	1.1	1.1	1.1	0.01	0.01	0.01
Annual subscription to EPA	10,000	10,000	10,000	0.5	0.5	0.5	0.01	0.01	0.01
Labour	279,136	279,136	279,136	14.5	14.9	15.0	0.17	0.18	0.18
Loan repayments (interest)	75,780	75,780	5780	3.9	4.0	4.1	0.05	0.05	0.05
Depreciation	175,021	175,021	175,021	.1	9.3	9.4	0.11	0.11	0.11
***Total fixed costs***	**665,117**	**665,117**	**665,117**	**34.7**	**35.4**	**35.8**	**0.42**	**0.43**	**0.43**
***Total costs***	**2,274,229**	**2,321,332**	**2,354,741**	**118.5**	**123.7**	**126.8**	**1.43**	**1.49**	**1.51**
***Net Profit***	**349,338**	**249,918**	**204,595**	**18.2**	**13.3**	**11.0**	**.22**	**0.16**	**0.13**

^a^A 728 sow farrow-to-finish farm with weekly farrowing batches was simulated to represent three different scenarios

^b^Scenario 1: a farrow-to-finish farm with prevalence of pleurisy < 25% and prevalence of lung scars < 8% with a wean-to-finish average daily gain (ADG) of 760 g and reaching target slaughter weight at 24 weeks of age

^c^Scenario 2: a farrow-to-finish farm with prevalence of pleurisy < 25% and prevalence of lung scars ≥8% (LPHSC) with an ADG of 725 g and reaching target slaughter weight at 25 weeks of age

^d^Scenario 3: a farrow-to-finish farm with prevalence of pleurisy ≥25% (HP) with and ADG of 671 g and reaching target slaughter weight at 26 weeks of age

Finally, when compared with the LPLSC farm, the LPHSC farm used 14.5% more weaner feed, 0.5% fewer finisher feed and sold 2.2% fewer pigs and 2.2% less meat (Table 2). Higher variable costs were observed in the LPHSC farm due to higher weaner feed costs, higher cost to produce replacement gilts and a greater number of dead animals for disposal. Sales were 2% lower in the LPHSC farm than in the LPLSC farm. Gross margin and net profit were 9.8 and 28.5% lower in the LPHSC farm compared with the LPLSC farm.

#### Risk analysis and risk assessment

**Fig. 4:**
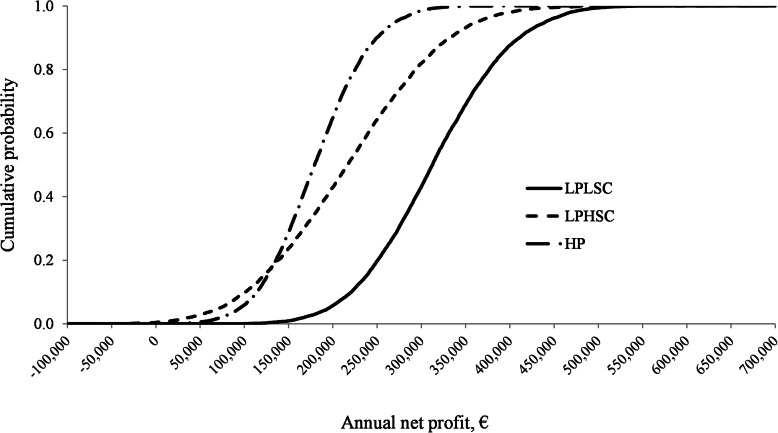
Cumulative distribution function showing the cumulative probability (*Y-*axis) of reaching a given level of profit (*X-*axis) for three scenarios simulating effects associated with different prevalence of pleurisy and lung scars on slaughter pigs on farm performance and profitability. LPLSC farms [farms with prevalence of pleurisy < 25% and prevalence of lung scars < 8% with a wean-to-finish average daily gain (ADG) of 760 g and reaching target slaughter weight at 24 weeks of age] were first-order stochastically dominant compared with LPHSC (farm with prevalence of pleurisy < 25% and prevalence of lung scars ≥8% (LPHSC) with an ADG of 725 g and reaching target slaughter weight at 25 weeks of age) and HP farms (farm with prevalence of pleurisy ≥25% (HP) with and ADG of 671 g and reaching target slaughter weight at 26 weeks of age). LPHSC farms were second-order stochastically dominant compared to HP farms. For all scenarios, 728 sow farrow-to-finish farms with weekly farrowing batches were simulated. A total of 10,000 iterations (i.e. farms) were simulated for each scenario by performing stochastic simulation analysis by Monte Carlo sampling using the Microsoft Excel add-in *@Risk* [44]

For the risk analysis and assessment 10,000 iterations were simulated. Results for the risk analysis are presented in Table 4 while results for the risk assessment are presented in Fig. 4. First-order stochastic dominance was observed for LPLSC farms over LPHSC and HP farms. Second-order stochastic dominance was observed for LPHSC over HP farms. Therefore, HP farms were more likely to lose money than LPHSC farms but they also had less volatile returns which implied that at some lower levels of profit LPHSC farms were expected to obtain slightly higher profits than HP farms (Fig. 4). Indeed, mean annual gross income decreased 13.5% and 4.2% HP farms compared with LPLSC and a LPHSC farms, respectively. A difference in gross margin of 9.6% was observed between LPLSC and LPHSC farms. Similarly, mean annual net profit decreased by 42.1% and 17.1% in HP farms compared with LPLSC and a LPHSC farms, respectively and mean annual net profit was 30.2% lower in the LHPSC farms compared with the LPLSC farms.

**Table 4:** Mean ± standard deviation (SD) and the associated 90% confidence interval (CI, 5 to 95%), minimum and maximum annual gross margin and net profit in farms^a^ with different prevalence of pleurisy and lung scars on slaughter pigs. Results were obtained during the risk analysis by performing stochastic simulation analysis by Monte Carlo sampling with 10,000 iterations (‘farms’) using the Microsoft Excel add-*in @Risk* [44]

Variable	€ per year			€ per pig			€ per kg		
	LPLSC^**b**^	LPHSC^**c**^	HP^**d**^	LPLSC	LPHSC	LP	LPLSC	LPHSC	HP
*Gross margin, €*									
Mean	978,020	883,579	846,131	51.1	47.2	45.2	0.61	0.57	0.54
SD	73,587	89,923	52,997	1.8	2.1	1.7	0.02	0.02	0.02
5% CI	861,044	740,681	761,039	48.1	43.8	42.4	0.58	0.53	0.51
95% CI	1,101,716	1,035,199	935,183	54.1	50.4	48.1	0.65	0.61	0.57
Minimum	730,977	571,026	657,281	43.7	38.0	38.2	0.53	0.46	0.46
Maximum	1,267,410	1,191,485	1,069,961	58.5	55.3	52.0	0.70	0.67	0.62
*Net profit, €*									
Mean	312,902	218,461	181,014	16.2	11.4	9.6	0.20	0.14	0.11
SD	73,587	89,923	52,997	3.1	4.1	2.6	0.04	0.05	0.03
90% CI	195,926-436,598	75,563-370,082	95,921-270,066	10.1–21.2	4.5–17.9	5.4–13.8	0.13–0.26	0.05–0.21	0.06–0.16
Minimum	65,860	−94,091	− 7836	4.0	−6.3	−0.5	0.05	−0.08	−0.01
Maximum	602,293	526,367	404,843	27.1	24.4	19.6	0.33	0.29	0.23

^a^728 sow farrow-to-finish farms with weekly farrowing batches were simulated to represent three different scenarios. A total of 10,000 iterations (i.e. farms) were simulated for each scenario. At each iteration, all stochastic input variables (i.e. biological inputs, feedstuff and pork prices) varied simultaneously by randomly sampling a new set of values for each variable from their corresponding distributions. Additionally, gross margins and net profit were calculated for each iteration

^b^Scenario 1: a farrow-to-finish farm with prevalence of pleurisy < 25% and prevalence of lung scars < 8% with a wean-to-finish average daily gain (ADG) of 760 g and reaching target slaughter weight at 24 weeks of age

^c^Scenario 2: a farrow-to-finish farm with prevalence of pleurisy < 25% and prevalence of lung scars ≥8% (LPHSC) with an ADG of 725 g and reaching target slaughter weight at 25 weeks of age

^d^Scenario 3: a farrow-to-finish farm with prevalence of pleurisy ≥25% (HP) with and ADG of 671 g and reaching target slaughter weight at 26 weeks of age

## Discussion

This study describes the main associations between pluck lesions recorded at slaughter and ADG in the grower-finisher period using regression trees. Then, these associations were used to simulate bio-economic performance of different types of farms defined by their types and levels of pluck lesions. The selected types and levels of lesions may not be the same for different cohorts of farms, but the methodology and the resulting economic consequences would be still of interest.

Pluck lesions are common findings in slaughtered pigs [9, 10, 47] and multiple lesions are often seen simultaneously [4, 11, 15]. Studying the association of different pluck lesions with key farm performance indicators is of interest to infer economic costs. Using linear models to study such associations is one of the options and probably the most classical approach. However, in practice, it is often needed to define cut-off points to implement a certain intervention (e.g. medication, vaccination, depopulation). These decisions can be done based on the veterinarian experience but there are several statistical tools that can help us to objectively estimate cut-off values. In the present study we propose the use of regression trees because they provide results in the format of a decision tree that are easily understood. With this approach, it is possible to obtain not only a hierarchization of the different factors affecting the output but also typologies of farms (i.e. scenarios) present within a cohort.

From the six recorded pluck lesions, pleurisy and lung scars were the main lesions associated with changes in ADG. We selected ADG as outcome because previous analyses of our database. We observed that ADG was negatively associated with respiratory disease by a greater extent than other key performance indicators such as feed conversion ratio (*R*^*2*^ *=* 40% vs. *R*^*2*^ *=* 14%, respectively for multivariable linear regression models with ADG or feed conversion ratio as predicted variables and pluck lesions as predictor variables, *unpublished data*). It is important to note that a causative relationship between these lesions and ADG is not suggested at any point although there is evidence of direct effects of pleurisy on performance [4, 7].

The levels of lesions were used in this study to classify farms and study their bio-economic performance beyond the direct effect in the grower-finisher stage. Both, pleurisy and scars are part of the PRDC. Pleurisy lesions in the dorso-caudal lobes are suggestive of *Actinobacillus pleuropneumoniae* [26, 48]*,* and scars are related to healed enzootic pneumonia caused by *Mycoplasma hyopneumoni*a. However, other pathogens are often involved in the development of such lesions [49, 50]. In fact, the PRDC includes pathogens that affect the herd in all the stages, like PRRS virus, and is also affected by management practices which are interrelated between stages at a farm level.

The estimated cut-off value for pleurisy in this study is lower than the alarm (55%; defined as the incidence above which a health plan is required) and warning (28% defined as the half value of an alarm value) cut-off values for pleurisy recommended by the Welfare Quality® Protocol for pigs [51] although these values were subjectively defined by consultation with experts from the animal science field. Also, our estimated cut-off value for pleurisy is higher than the one selected by Jäger et al. [2] of < 5% (low prevalence) and > 10% (high prevalence). The different pleurisy cut-off values found between studies may be attributable to the different methods, sample population and year when data was obtained for their estimation. We used an objective method (i.e. regression tree analysis) to estimate the cut-off values while Jäger et al. [2] subjectively chose the cut-off values. Jäger et al. [2] monitored farms participating in the BPHS abattoir pathology monitoring scheme database and that had at least 50 pigs assessed in at least three occasions in 24 months. Then they subjectively decided on cut-off values based on examining the distribution of the full dataset and on a sample size calculation to determine the minimum number of farms needed in each classification.

Jäger et al. [2] reported increased weaner mortality in farms with higher prevalence of pleurisy and Jäger et al. [21] estimated a pleurisy cost of £5 (approximately €5.8) based on industry standard values. This is lower than the cost of €7.9 per pig produced estimated in this study when compared to pigs produced under a scenario considering a farm with lower prevalence of pleurisy and lung scars. Direct comparison with our results is however difficult as Jäger et al. [21] based their calculation on an increased mortality rate of 1% being worth 50p per pig and considering a rise in mortality from 2 to 8% being worth £3 per pig. They also estimated that for a batch of 100 pigs with a prevalence of pleurisy of 20% and a reduction in ADG of 50 g, the cost will be £21 across the batch (21p per pig). To this they summed a cost of £1.40 per pig for a change of 0.1 in feed conversion efficiency plus costs associated with medication and labour for treatment. Furthermore, feed costs differed between studies; Jäger et al. [21] assumed feed costs of £140/t for all kinds of feed provided during the grower-finisher period while we used current market values and thus, feed cost varied in the different production stages. Therefore, assumptions for economic analysis were different between the study by Jäger et al. [21] and the current study.

Under the conditions of this study, a farm with a high prevalence of pleurisy (≥ 25%) will experience greater economic loses than a farm with pleurisy at a lower prevalence even when combined with high prevalence of other lung lesions such as lung scars (> 8%). Under the HP scenario, a farm would experience higher variable costs, mainly due the associated lower ADG during the grower-finisher period which would result in increased weaner feed usage as pigs would require an extra week in the second weaner stage to reach adequate weight for transfer (i.e. 38 kg of body weight) to the finisher stage. Also, a farm under the HP scenario would use more finished feed as pigs would also require more time in the finisher stage to reach target slaughter weight (i.e. 110.8 kg of body weight); this would increase finisher feed costs. Furthermore, the HP scenario was associated with higher mean mortality rates throughout the production cycle which would increase carcass disposal costs. Also, higher mortality rates in the HP scenario would decrease the number of pigs and kg of meat produced thereby reducing farm income. When examining results from the risk analysis, farms simulated under the HP scenario were less profitable, at any level, than farms simulated under the LPLSC or LPHSC scenarios. For example, HP farms only had 10% probability of reaching an annual net profit of €250,000 while the probability of reaching the same annual net profit was 81% for LPLSC farm and 36% for LPHSC farms. These findings confirm that better lung health is economically advantageous and suggest that emphasis should be placed on improving animal health as means to improve performance.

Our results also suggest that a farm where the prevalence of pleurisy is low, a higher prevalence of lung scars would result in greater economic risk. In this study, pigs produced under the LPHSC scenario would require one extra week to be transferred from the weaner to the finisher stage which would increase weaner feed usage and costs. Other variables cost would also increase in a LPHSC farm. For example, under the LPHSC scenario, a farm would have higher replacement gilt costs because sow replacement rate (i.e. sow culling and sow mortality rates) was higher than in LPLSC scenario farm and it would require to produce more replacement gilts per week to maintain breeding herd numbers. We cannot directly attribute the higher sow replacement rate in the LPHSC scenario farm to the higher prevalence of lung scars and thus, we are unable to provide an explanation for this result. Sows are culled/die due to several causes and thus, this result should be viewed with caution.

Some methodological aspects of the current study are worth considering when interpreting the results. For instance, farms were invited to participate in the study based on the criteria of providing performance records to the Teagasc e-Profit Monitor system. Previous work reported differences between farms recordkeeping in the Teagasc e-Profit Monitor and those that do not participate in the Teagasc e-Profit monitor with regards to welfare indicators [52] and thus, it is possible that farms agreeing to participate in this study had better health and performance when compared to farms not participating in the Teagasc e-Profit Monitor. However, the 107 herds providing data to the Teagasc e-Profit Monitor during the year 2017 represented 53% of the national sow herd and a high participation rate (52%) was obtained among the farmers contacted. Moreover, key performance indicators were similar between participating and non-participating farms recordkeeping in the Teagasc e-Profit Monitor [43]. Therefore, the 56 participating farms provide a representative sample of all herds in the Teagasc e-Profit Monitor, and constitute a large sample of 29.2% of the Irish national sow herd.

The economic calculations presented in this study were based on mean values for a cohort of farms representing different prevalence of pleurisy and lung scars associated with changes in ADG. Thus, we also acknowledge that the biological assumptions used to parameterise the three scenarios investigated in this study were likely affected by other diseases and many other management and environmental issues present on the farms. Integration of other diseases and confounding effects is very complex and introduces more uncertainty around the contribution of different diseases/environmental issues on key biological parameters affected [53]. However using performance data from real farms takes into account the multifactorial nature of different pathogens/environmental issues and their associated lesions. Changes in the prevalence of a specific pathogen or changes in environmental conditions (e.g. animal husbandry practices) would result in changes in the prevalence of a secondary pathogen as they interact among them. Thus, it is questionable if the impact of a single pathogen or environmental condition can be truly isolated from each other [53]. Moreover, monitoring several batches of pigs at regular time intervals will provide a more reliable measure of the prevalence of different pluck lesions on a given farm compared with using the information of a single batch as in this study; however, it is not unusual to determine the prevalence of pluck lesions based on a single visit to the abattoir in countries without computerized systems for lesions recording (e.g. [11, 26]).

In this study we decided to use the prevalence of lesions, instead of the prevalence of different severity of lesions (in the case of pleurisy and pneumonia), as we were interested in identifying which lesions, and their possible combinations, were more associated with changes in ADG. By using the prevalence of different scores, it would be likely that the regression tree results presented cut-off values for different severities for the same lesions e.g. pleurisy since it was the main lesion associated with changes in ADG. Associations between ADG and prevalence of different severity scores for e.g. pleurisy and their bio-economic simulation would indeed be interesting for a follow up study. Also, while we accounted for differences in ADG, additional costs from e.g., differences in condemnation rates associated with a different prevalence of pleurisy and lung scars lesions, or different management strategies, labour requirements, and healthcare treatments to reduce these lesions were not available for inclusion in the TPPM for the economic analysis due to a lack of data. We also acknowledge that a longer grower-finish period as per the HP and LPHSC scenarios would require extra space to accommodate the pigs and that we did not account for it in the bio-economic simulation. However, in reality farmers are unable to provide additional housing to accommodate pigs for longer because of higher prevalence of pluck lesions. Farmers are likely to focus on management strategies that could improve ADG and/or that would lower prevalence of the lesions in the long term, thereby reducing the time pigs require to reach target slaughter weight. Further expansion of the TPPM to include these additional aspects would be a natural continuation for this study once the necessary information becomes available. This will further aid in determining the cost implications of management strategies currently used, as well as the feasibility of implementing new practices to improve animal health and performance.

## Conclusion

Under the conditions of this study, higher prevalence of pleurisy and lung scars were associated with decreased ADG during the grower-finisher period and with lower economic return in the simulated farms. The regression tree analysis illustrates an easy to understand objective method that could be used by pig producers and veterinary practitioners to identify cut-off points for the prevalence of various health problems to decide when to implement disease prevention/eradication strategies on their farms to minimise adverse disease effects on animal performance. A lower prevalence of lung lesions was associated with lower economic risk at any given level of profit confirm that better lung health is economically advantageous. Thus, our results also form a foundation for future work which should consider additional variables related to control of pluck lesions on pig farms and longitudinal approaches. Additionally, our results highlight the economic benefits and importance of preventing or controlling respiratory disease.

## Data Availability

The R code used for the regression tree analysis and datasets for the results presented in this study are available from the corresponding author upon reasonable request.

## References

[1] VanAlstine W, Zimmerman J, Karriker L, Ramirez A, Schwartz KJ, Stevenson G (2010). Respiratory system. Dis Swine.

[2] Jäger HC, McKinley TJ, Wood JLN, Pearce GP, Williamson S, Strugnell B (2012). Factors associated with pleurisy in pigs: a case-control analysis of slaughter pig data for England and Wales. PLoS One.

[3] Scollo A, Gottardo F, Contiero B, Mazzoni C, Leneveu P, Edwards SA. Benchmarking of pluck lesions at slaughter as a health monitoring tool for pigs slaughtered at 170 kg (heavy pigs). Prev Vet Med. 2017;144:20–28. 10.1016/j.prevetmed.2017.05.007. 10.1016/j.prevetmed.2017.05.007 28716200

[4] Pagot E, Pommier P, Keïta A (2007). Relationship between growth during the fattening period and lung lesions at slaughter in swine. Rev Med Vet (Toulouse).

[5] Greve J h. Internal parasites: Helminths. In: Zimmerman JJ, Karriker LA, Ramirez A, Schwartz KJ, Stevenson GW, editors. Dis Swine 10th ed. 10th editi. Oxford, UK: Wiley-Blackwell; 2012. p. 908–920.

[6] Ferraz MES, Almeida HMS, Storino GY, Sonálio K, Souza MR, Moura CAA, et al. Lung consolidation caused by mycoplasma hyopneumoniae has a negative effect on productive performance and economic revenue in finishing pigs. Prev Vet Med 2020;182:105091. 10.1016/j.prevetmed.2020.105091. 10.1016/j.prevetmed.2020.105091 32683190

[7] Straw BE, Shin SJ, Yeager AE (1990). Effect of pneumonia on growth rate and feed efficiency of minimal disease pigs exposed to Actinobacillus pleuropneumoniae and mycoplasma hyopneumoniae. Prev Vet Med..

[8] Fraile L, Alegre A, López-jiménez R, Nofrarías M, Segalés J. Risk factors associated with pleuritis and cranio-ventral pulmonary consolidation in slaughter-aged pigs. Vet J 2010;184:326–333. 10.1016/j.tvjl.2009.03.029. 10.1016/j.tvjl.2009.03.029 19527939

[9] Meyns T, Van Steelant J, Rolly E, Dewulf J, Haesebrouck F, Maes D. A cross-sectional study of risk factors associated with pulmonary lesions in pigs at slaughter. Vet J 2011;187:388–392. 10.1016/j.tvjl.2009.12.027. 10.1016/j.tvjl.2009.12.027 20122861

[10] Martínez J, Peris B, Gómez EA, Corpa JM (2009). The relationship between infectious and non-infectious herd factors with pneumonia at slaughter and productive parameters in fattening pigs. Vet J.

[11] Fablet C, Marois C, Dorenlor V, Eono F, Eveno E, Jolly JP, et al. Bacterial pathogens associated with lung lesions in slaughter pigs from 125 herds. Res Vet Sci 2012;93:627–630. 10.1016/j.rvsc.2011.11.002. 10.1016/j.rvsc.2011.11.002 22133708

[12] Steenhard NR, Jungersen G, Kokotovic B, Beshah E, Dawson HD, Urban JF (2009). Ascaris suum infection negatively affects the response to a mycoplasma hyopneumoniae vaccination and subsequent challenge infection in pigs. Vaccine.

[13] Maes D, Verdonck M, Deluyker H, de Kruif A (1996). Enzootic pneumonia in pigs. Vet Q.

[14] Roepstorff A, Eriksen L, Slotved H, Nansen P (1997). Experimental Ascaris suum infection in the pig: worm population kinetics following single inoculations with three doses of infective eggs. Parasitology.

[15] Brewster VR, Maiti HC, Tucker AW, Nevel A. Associations between EP-like lesions and pleuritis and post trimming carcass weights of finishing pigs in England. Livest Sci 2017;201:1–4. 10.1016/j.livsci.2017.04.012.

[16] Vlaminck J, Düsseldorf S, Heres L, Geldhof P. Ascaris suum , lung pathogens and technical performance parameters. Vet Parasitol 2015;210:151–158. 10.1016/j.vetpar.2015.04.012. 10.1016/j.vetpar.2015.04.012 25952722

[17] Cleveland-Nielsen A, Nielsen EO, Ersboll AK (2002). Chronic pleuritis in Danish slaughter pig herds. Prev Vet Med.

[18] Teixeira DL, Harley S, Hanlon A, Connell NEO, Dwyer CM (2016). Study on the association between tail lesion score, cold carcass weight, and viscera condemnations in slaughter pigs. Front Vet Sci.

[19] Niemi JK, Jones P, Tranter R, Heinola K. Cost of production diseases to pig farms. 24th Int Pig Vet Soc Congr. Dublin, Ireland; 2016. p. 302.

[20] Stygar AH, Niemi JK, Oliviero C, Laurila T, Heinonen M. Economic value of mitigating Actinobacillus pleuropneumoniae infections in pig fattening herds. Agric Syst 2016;144:113–121. 10.1016/j.agsy.2016.02.005.

[21] Jäger HJ, Mckinley TJ, Pearce GP, Tucker AW, Wood JLN. Pleurisy in Pigs : Associated risk factors and impact on health , welfare. BPEX Rep. 2009:1–94..

[22] Kragt ME. Bioeconomic modelling: Integrating economic and environmental systems? 2012 Int Congr environ model Softw Manag Resour a ltd planet, Sixth Bienn Meet 2012;9. http://www.iemss.org/sites/iemss2012/proceedings/D8_0464_Kragt.pdf.

[23] Marquer P, Rabade T, Forti R. Pig farming in the European Union: considerable variations from one member state to another. 2014.

[24] Hansen MS, Pors SE, Jensen HE, Bille-Hansen V, Bisgaard M, Flachs EM (2010). An investigation of the pathology and pathogens associated with porcine respiratory disease complex in Denmark. J Comp Pathol.

[25] Bochev I (2007). Porcine respiratory disease complex ( Prdc ): a review. I. etiology , epidemiology , clinical forms and pathoanatomical features. Bulg J Vet Med.

[26] Jirawattanapong P, Stockhofe-Zurwieden N, van Leengoed L, Wisselink H, Raymakers R, Cruijsen T, et al. Pleuritis in slaughter pigs: relations between lung lesions and bacteriology in 10 herds with high pleuritis. Res Vet Sci 2010;88:11–15. 10.1016/j.rvsc.2009.06.007. 10.1016/j.rvsc.2009.06.007 19836811

[27] Madec F, Derrien H. Frequence, intensite et localosation des lesion pulmonaires chez le porc charcutier. 13es Journées la Rech Porc. Paris, France; 1981. p. 231–6.

[28] Christensen G, Soarensen V, Mousing J. Diseases of the respiratory system. In: Straw B, D’Allaire S, Taylor DJ WM, editors. Dis Swine. 8th ed. 1999. p. 913–40.

[29] Ostanello F, Dottori M, Gusmara C, Leotti G, Sala V (2007). Pneumonia disease assessment using a slaughterhouse lung-scoring method. J Vet Med Ser A Physiol Pathol Clin Med.

[30] Calderón Díaz JA, Shalloo L, Niemi JK, Kyriazakis I, Mckeon M, Mccutcheon G (2019). Description, evaluation, and validation of the teagasc pig production model. J Anim Sci.

[31] Breiman L, Friedman J, Olshen R, Stone C (1984). Classification and regression trees.

[32] Strobl C, Malley J, Tutz G (2009). An introduction to recursive partitioning: rationale, application and characteristics of classification and regression trees, bagging and random forests. Psychol Methods.

[33] Speybroeck N, Berkvens D, Mfoukou-Ntsakala A, Aerts M, Hens N, Van Huylenbroeck G (2004). Classification trees versus multinomial models in the analysis of urban farming systems in Central Africa. Agric Syst.

[34] Morgan J. Classification and regression trees. Tech. Rep. Boston; 2014.

[35] Lewis RJ, Ph D, Street WC. An Introduction to Classification and Regression Tree (CART ) Analysis. 2000 Annu Meet Soc Acad Emerg Med. 2000. p. 14p. Available from: http://citeseerx.ist.psu.edu/viewdoc/download?doi=10.1.1.95.4103&amp;rep=rep1&amp;type=pdf.

[36] Lemon SC, Roy J, Clark MA, Friedmann PD, Rakowski W (2003). Classification and regression tree analysis in public health: methodological review and comparison with logistic regression. Ann Behav Med.

[37] Krzywinski M, Altman N (2017). Classification and regression trees. Nat Methods.

[38] Yang L, Liu S, Tsoka S, Papageorgiou LG. A regression tree approach using mathematical programming. Expert Syst Appl 2017;78:347–357. 10.1016/j.eswa.2017.02.013.

[39] Therneau T, Atkinson B. rpart: Recursive Partitioning and Regression Trees. 2018. https://cran.r-project.org/package=rpart%0A.

[40] R Core Team (2017). R: a language and environment for statistical computing.

[41] Wellock I, Emmans G, Kyriazakis I (2004). Describing and predicting potential growth in the pig. Anim Sci.

[42] National Research Council (2012). Nutrient requirements of swine.

[43] Teagasc. National Pig Herd Performance Report - 2017. 2018.

[44] Palisade (2013). @Risk Risk Analysis Add-in for Microsoft Excel Versions 6.1.2.

[45] Strong RA, Steiger NM, Wilson JR. Introduction to financial risk assessment using Monte Carlo simulation. Austin: Winter Simulation Conference; 2009. p. 99-118.

[46] Hardaker JB, Lien G, Anderson JR, Huirne RBM (2015). Coping with risk in agriculture: applied decision analysis. 3rd. Editi.

[47] Maes D, Chiers K, Haesebrouck F, Laevens H, Verdonck M, De Kruif A (2001). Herd factors associated with the seroprevalences of Actinobacillus pleuropneumoniae serovars 2,3 and 9 in slaughter pigs from farrow-to-finish pig herds. Vet Res.

[48] Merialdi G, Dottori M, Bonilauri P, Luppi A, Gozio S, Pozzi P (2012). Survey of pleuritis and pulmonary lesions in pigs at abattoir with a focus on the extent of the condition and herd risk factors. Vet J.

[49] Villarreal I, Meyns T, Dewulf J, Vranckx K, Calus D, Pasmans F (2011). The effect of vaccination on the transmission of mycoplasma hyopneumoniae in pigs under field conditions. Vet J.

[50] Holt HR, Alarcon P, Velasova M, Pfeiffer DU, Wieland B (2011). BPEX pig health scheme: a useful monitoring system for respiratory disease control in pig farms?. BMC Vet Res.

[51] Welfare Quality Consortium (2009). Welfare Quality® assessment protocol for pigs.

[52] Van Staaveren N, Teixeira DL, Hanlon A, Boyle LA (2017). Pig carcass tail lesions: the influence of record keeping through an advisory service and the relationship with farm performance parameters. Animal.

[53] Nathues H, Alarcon P, Rushton J, Jolie R, Fiebig K, Jimenez M, et al. Cost of porcine reproductive and respiratory syndrome virus at individual farm level – an economic disease model. Prev Vet Med 2017;142:16–29. 10.1016/j.prevetmed.2017.04.006. 10.1016/j.prevetmed.2017.04.006 28606362

